# Soil Fungal Community Structure and Function Shift during a Disease-Driven Forest Succession

**DOI:** 10.1128/spectrum.00795-22

**Published:** 2022-09-08

**Authors:** Zhao-lei Qu, Ahmed Braima, Bing Liu, Yang Ma, Hui Sun

**Affiliations:** a Collaborative Innovation Center of Sustainable Forestry in Southern China, College of Forestry, Nanjing Forestry University, Nanjing, China; b Yangzhou Polytechnic College, Yangzhou, China; Yeungnam University

**Keywords:** fungal community, forest succession, enzyme activity, pine wilt disease, microbe-plant interactions

## Abstract

Forest succession is important for sustainable forest management in terrestrial ecosystems. However, knowledge about the response of soil microbes to forest disease-driven succession is limited. In this study, we investigated the soil fungal biomass, soil enzyme activity, and fungal community structure and function in forests suffering succession processes produced by pine wilt disease from conifer to broadleaved forests using Illumina Miseq sequencing coupled with FUNGuild analysis. The results showed that the broadleaved forest had the highest fungal biomass and soil enzyme activities in C, N, and S cycles, whereas the conifer forest had the highest enzyme activity in the P cycle. Along the succession, the fungal diversity and richness significantly increased (*P *< 0.05). The fungal communities were dominated by Ascomycota (42.0%), Basidiomycota (38.0%), and Mortierellomycota (9.5%), among which the abundance of Ascomycota significantly increased (*P *< 0.05), whereas that of Basidiomycota and Mortierellomycota decreased (*P *< 0.05). The abundance of species Mortierella humilis, Lactarius salmonicolor, and Russula sanguinea decreased, whereas that of Mortierella minutissima increased (*P *< 0.05). The forests in different succession stages formed distinct fungal communities and functional structures (*P *< 0.05). Functionally, the saprotrophs, symbiotrophs, and pathotrophs were the dominant groups in the conifer, mixed, and broadleaved forests, respectively. Soil pH and soil organic carbon were the key factors influencing the fungal community and functional structures during the succession. These findings provide useful information for better understanding the plant-microbe interaction during forest succession caused by forest disease.

**IMPORTANCE** The studies on soil fungal communities in disease-driven forest succession are rare. This study showed that during the disease-driven forest succession, the soil enzyme activity, soil fungal diversity, and biomass increased along succession. The disease-driven forest succession changed the soil fungal community structure and function, in which the symbiotrophs were the most dominant group along the succession. These findings provide useful information for better understanding the plant-microbe interaction during forest succession caused by forest disease.

## INTRODUCTION

Forest succession caused by natural disturbance can affect a wide array of terrestrial ecosystem processes and is important for sustainable forest management (1). The ecological linkages between aboveground and belowground biota during forest succession have been considered an important mechanism in forest development and succession (2). For example, wildfire disturbance can change the forest soil chemistry and soil enzyme activity, which reduces the diversity and abundance of mycorrhizal fungi (3). The plant changes caused by succession can affect the soil microbial community, which, in turn, can regulate the plant growth via moderating the decomposition of soil organic matter (SOM) (4). Many studies have focused on the effects of forest succession on the aboveground plant community assembly (5), tree architecture variability (6), and plant nutrient use strategies (7), but knowledge on the dynamics of soil microbial communities and their functions during the disease-driven forest succession process is limited.

Soil microbes generally have rapid responses and high turnover rates in response to environmental disturbance, which could provide additional information on the forest succession mechanism; for example, plant species can strongly affect the soil microbial community structure and functions through the allocation of plant carbon and other nutrients (8, 9). Soil abiotic properties, including pH and nutrient availability, are key regulators that link plant performance with soil microbial communities (10, 11). Soil fungi represent an essential functional component of soil as decomposers, symbionts, and pathogens, in which saprophytic soil fungi can decompose soil substrates and increase the soil nutrient cycle, while ectomycorrhizal fungi, such as symbiotrophs, are known to enhance the nutritional condition of plants (12, 13). Fungal biomass and microbial enzyme activity are sensitive to changes in soil characteristics (14). In general, the deciduous forest has significantly higher soil fungal biomass when compared with that of the conifer forest (15). The extracellular soil enzymes produced by fungi can be involved in soil carbon (C), nitrogen (N), and phosphorus (P) cycles, which have a significant effect on the initial decomposition of plant litter (16, 17). Many environmental factors, such as plant species, can influence soil enzyme activities (18, 19). Therefore, soil enzyme activities and the fungal biomass are often used as indicators to reflect changes in the ecosystem due to their rapid response to environmental changes.

Forest decline has contributed to changes in soil nutrients and properties. The most recent outbreak caused by mountain pine beetle (Dendroctonus ponderosae) has affected more than 14 million hectares of forest land and triggered forest decline in western Canada (20). Compared with undisturbed stands, beetle-killed lodgepole pine (Pinus contorta var. *latifolia* Engelm.) stands have caused a decrease in soil phenolic content and increased soil moisture content and available nutrients (21, 22). Increased soil phenolics have led to decreased colonization rates in ectomycorrhizal fungi (23). Soil phenolics belong to a class of carbon-rich plant secondary compounds and are known to affect nutrient availability, especially nitrogen (23, 24). A previous study has demonstrated a decreased richness of ectomycorrhizal fungi in beetle-killed pine stands (25). However, there is a lack of research on the response of soil microbes to forest decline induced by forest disease.

Pine wilt disease (PWD) caused by pinewood nematode (PWN) Bursaphelenchus xylophilus is one of the most serious diseases worldwide, affecting several species of pine trees (*Pinus* spp.) and resulting in huge economic and environmental losses (26–28). PWD was first discovered on Japanese black pine (Pinus thunbergii Parl.) in China in 1982 in Purple Mountain Park, Nanjing, China, where the vegetation type is a pure conifer forest established in the late 1970s (29, 30). Since then, the disease has rapidly spread in China and continuously killed conifer trees. Currently, the forest in Nanjing Zijin Mountain Park is still undergoing forest succession from conifer of *P. thunbergii* and Pinus massoniana to deciduous trees of Liquidambar formosana (Sweetgum) and *Quercus* spp. (Oak) due to the occurrence of PWD. In the current study, we selected three typical forest types representing the entire forest succession process from initial conifer forest to intermediate mixed forest and eventual pure broadleaved forest. The three forests include a pure forest (*P. thunbergii*), a mixed pine and broadleaved forest (*P. thunbergia* + *L*. *formosana*), and a pure broadleaved forest (*L*. *formosana*). The intermediate mixed forest is defined by the equal ratio (1:1) between *P. thunbergii* and *L*. *formosana* trees (31). The eventual pure broadleaved forest is defined by the last pine tree being recently removed due to PWD. As PWD is the only and continuous driving force for forest decline and subsequent succession, our hypothesis is that the response of soil microbes in such a process might differ from those in which the driving forces are transient, such as fire, storm, or lightning. Therefore, the aims of the study are (i) to elucidate the changes in soil enzyme activity and fungal biomass during PWD-induced forest succession, (ii) to investigate the response of the soil fungal community structure and function, and (iii) to determine the environmental factors contributing to the dynamic changes in the fungal community during forest succession. To our best knowledge, there is no information available on the dynamic responses of soil microbes to forest succession caused by forest disease.

## RESULTS

### Soil fungal biomass and enzyme activity during forest succession.

**FIG 1:**
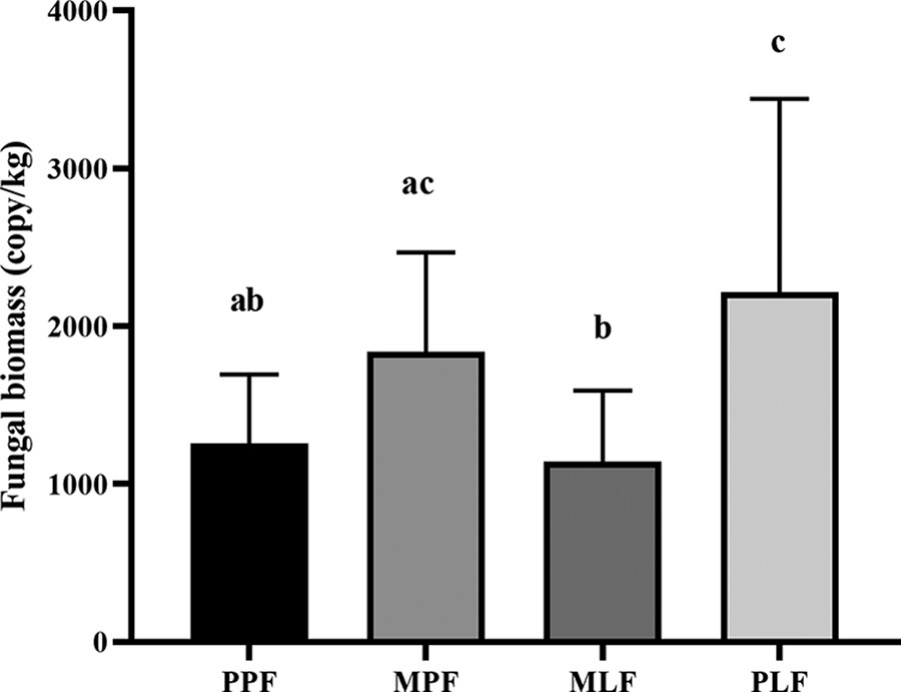
Soil fungal biomass in pure *P. thunbergii* forest (PPF), mixed forest (MPF + MLF), and pure *Liquidambar* forest (PLF) during forest succession. Letters in the figure show the significant difference (*P *< 0.05) among forest types.

The soil fungal biomass differed among forest sites (Fig. 1). The pure *L*. *formosana* forest (pure *Liquidambar* forest [PLF]) had the highest fungal biomass, which differed from that in the pure pine forest (PPF) and mixed forest (mixed *Liquidambar* forest [MLF]) (*P* < 0.05), whereas the MLF had the lowest fungal biomass. In the mixed forest, the fungal biomass of the mixed pine forest (MPF) was significantly higher than that of MLF (*P* < 0.05).

The soil enzyme activity of β-xylosidase (XYL), β-cellobiosidase (CEL), and β-glucosidase (GLS) involved in the C cycle; phosphatase (PHO) involved in the P cycle; and sulfatase (SUL) involved in the S cycle differed among sites (Table 1). The pure pine forest had the lowest enzyme activities of CEL, GLS, and SUL, which increased during forest succession (*R*^2^ = 0.9324, *R*^2^ = 0.7895, and *R*^2^ = 0.6078, respectively). The activity of PHO was the highest in the PPF site and differed significantly from that in other sites (*P* < 0.05). The activity of *N*-acetylglucosaminidase (NAG) involved in the N cycle and β-d-glucuronidase (GLR) involved in the C cycle increased (*R*^2^ = 0.9466 and *R*^2^ = 0.8883, respectively) during the succession.

**TABLE 1:** The soil enzyme activities in the three forests during forest succession^*a*^

Forest type	C cycle				N cycle NAG	P cycle PHO	S cycle SUL
	XYL	GLR	CEL	GLS			
PPF	16.29 ± 1.74AB	3.12 ± 0.29A	23.91 ± 2.47B	64.52 ± 5.25B	67.73 ± 6.01A	505.89 ± 31.1A	29.04 ± 5.1B
MPF	9.86 ± 1.25B	5.01 ± 1.28A	38.35 ± 5.06B	109.03 ± 15.89B	70.61 ± 7.62A	260.68 ± 24.03B	35.98 ± 7.11B
MLF	16.01 ± 1.72AB	5.77 ± 0.49A	46.97 ± 5.85B	96.36 ± 16.54B	78.21 ± 7.17A	266.55 ± 50.06B	30.22 ± 5.85B
PLF	18.26 ± 2.34A	6.14 ± 0.87A	77.61 ± 9.45A	167.17 ± 17.12A	88.42 ± 10.84A	365.8 ± 33.34B	121.11 ± 22.18A

a The data are presented as the mean ± standard deviation. The different letters in the table indicate significant difference (*P *< 0.05) among forest types. PPF, pure *P*. *thunbergii* forest; MPF, mixed *P. thunbergii* forest; MLF, mixed *L*. *formosan* forest; PLF, pure *L*. *formosan* forest; XYL, β-xylosidase; GLR, β-d-glucuroniase; CEL, β-cellobiosidase; GLS, β-glucosidase; NAG, N-acetylglucosaminidase; PHO, phosphatase; SUL, sulfatase.

### Fungal community α-diversity during forest succession.

**FIG 2:**
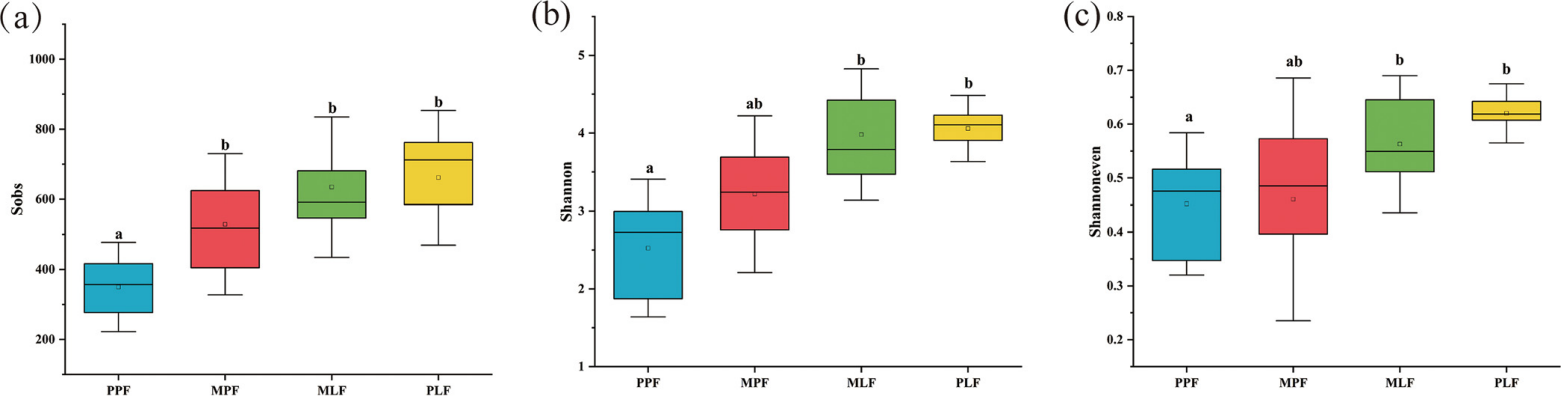
Fungal community richness (a), α-diversity (b), and evenness (c) in pure *P. thunbergii* forest (PPF), mixed forest (MPF + MLF), and pure *Liquidambar* forest (PLF) during forest succession. Letters in the figure show the significant difference (*P *< 0.05).

In total, 5,664 operational taxonomic units (OTUs) were estimated across all samples. The PPF had the lowest OTU richness (349 ± 99), α-diversity (2.71 ± 0.65), and evenness (0.46 ± 0.09), among which, the OTU richness significantly differed from that in mixed and PLF sites (*P* < 0.05). The PLF site had significantly higher α-diversity, richness, and evenness than the PPF site (*P* < 0.05). Interestingly, the diversity index did not differ in the mixed forest between the pine and broadleaved tree species (Fig. 2).

### Fungal community structure at the taxonomic level during forest succession.

**FIG 3:**
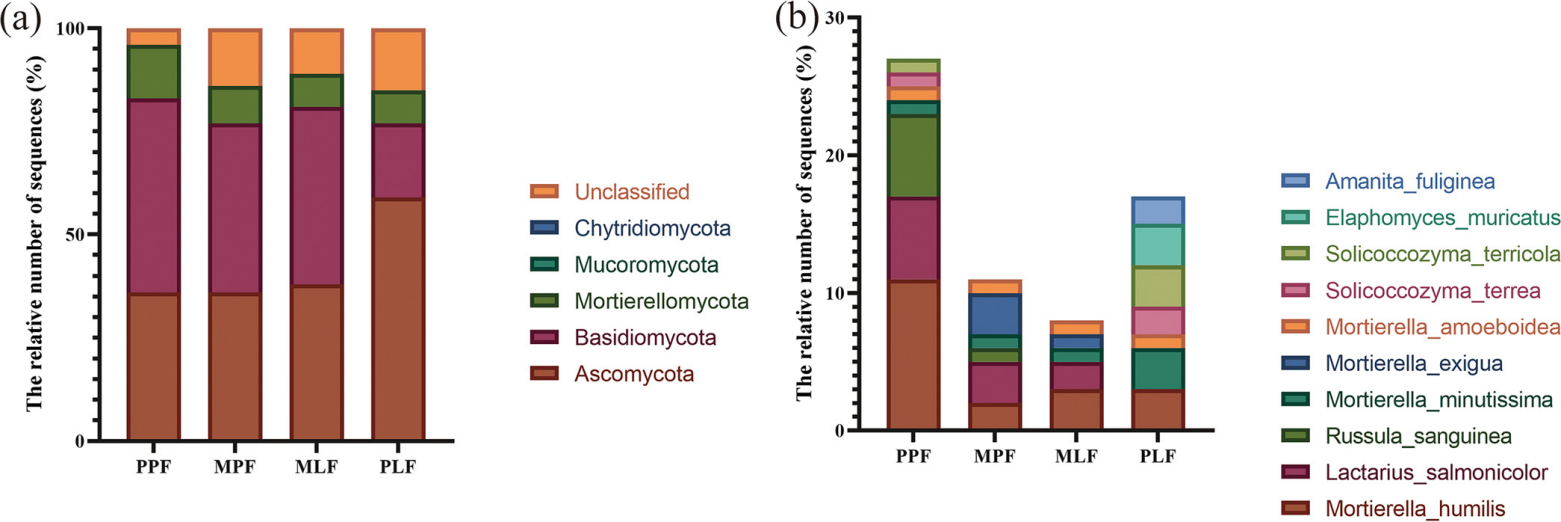
(a) The most abundant fungal groups at the phylum level. (b) The top 10 species in pure *P. thunbergii* forest (PPF), mixed forest (MPF + MLF), and pure *Liquidambar* forest (PLF) during forest succession.

All sequences were classified into the fungi domain, including 5 phyla, 12 classes, 20 genera, and 30 species (see Fig. S1 in the supplemental material). Ascomycota was the dominant phylum (42.0%), followed by Basidiomycota (38.0%), Mortierellomycota (9.5%), Mucoromycota (0.2%), and Chytridiomycota (0.1%) (Fig. 3).

The abundances of Ascomycota were the highest in the PLF site, which had the lowest abundance of Basidiomycota (Fig. 3a). Basidiomycota was more abundant in the PPF site. The abundance of Mortierellomycota was also the highest in the PPF site, which differed significantly from that in other sites (*P* < 0.05) (Fig. 3a). *Mortierella* was the most abundant genus at the genus level, followed by *Penicillium*, *Russula*, *Lactarius*, *Clavulina*, *Trichoderma*, *Sebacina*, *Geminibasidium*, *Solicocozyma*, and *Sistotrema* (see Fig. S2 in the supplemental material).

Mortierella humilis was the most abundant species at the species level, followed by Lactarius salmonicolor, Russula sanguinea, and Mortierella minutissima (Fig. 3b). Compared to the MPF site, the PPF site had higher abundances of *Mortierella humilis*, *Lactarius salmonicolor*, and *Russula sanguinea* but a lower abundance of Mortierella exigua. In the *Liquidambar* forest, the PLF had a higher abundances of *Mortierella minutissima*, Solicoccozyma terricola, Amanita fuliginea, and Elaphomyces muricatus than the mixed MLF. The MPF had a higher abundance of *Mortierella exigua* than the MLF site (Fig. 3b).

### Fungal community structure at OTU level during forest succession.

**FIG 4:**
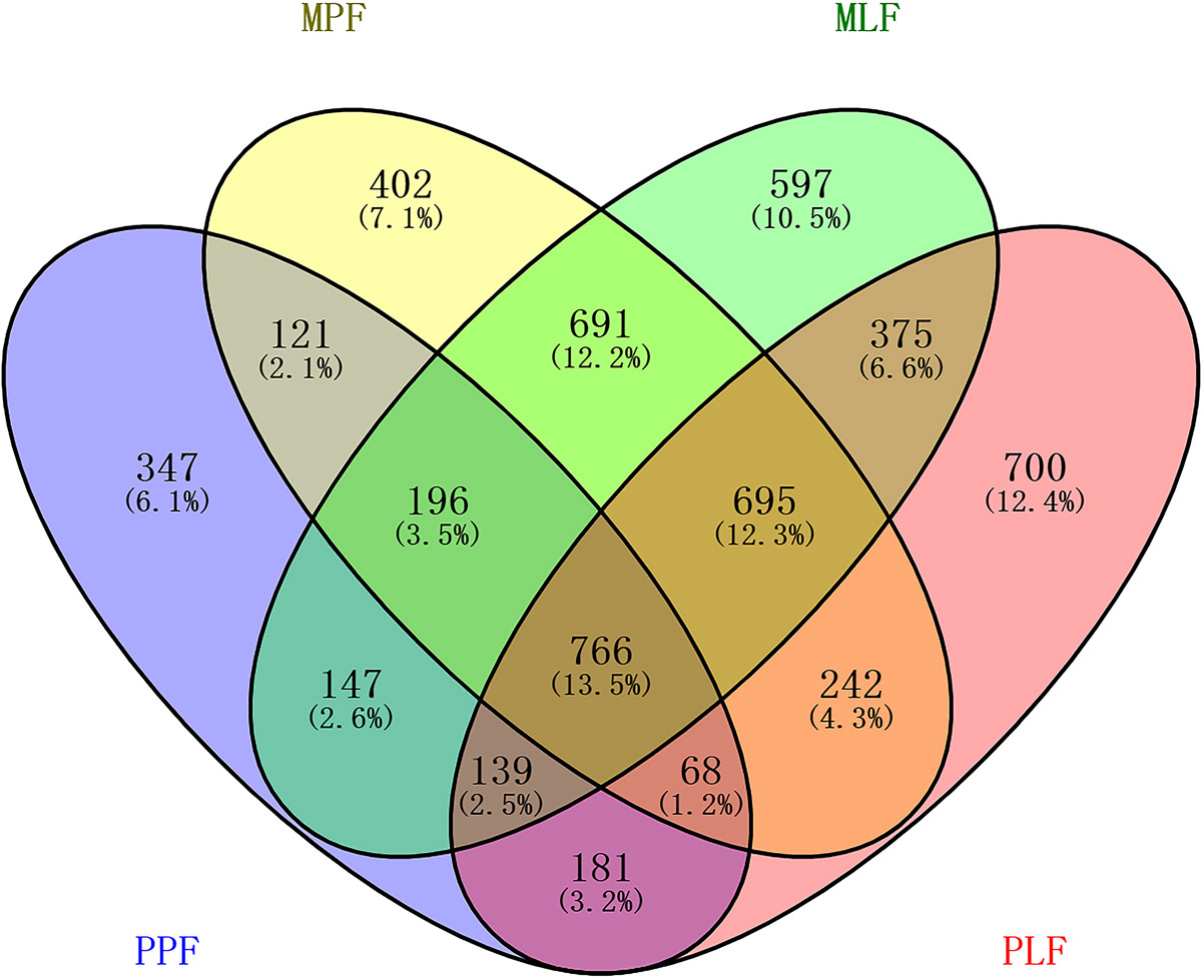
Venn diagrams showing the unique and shared fungal OTUs among pure *P. thunbergii* forest (PPF), mixed forest (MPF + MLF), and pure *Liquidambar* forest (PLF) during forest succession.

**FIG 5:**
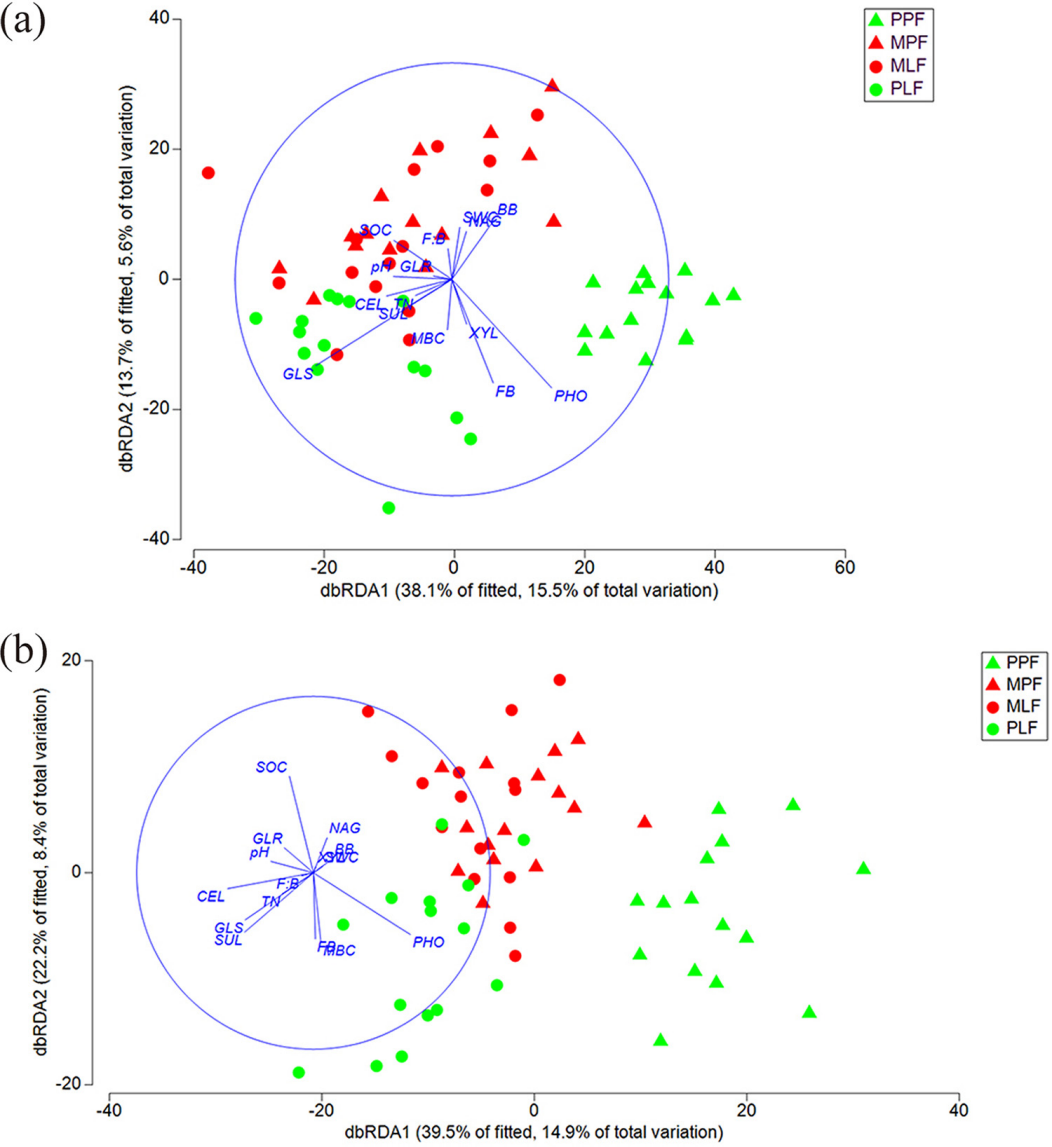
Distance-based redundancy analysis (dbRDA) showing fungal community structure (a) and functional structure (b) in the three forests among pure *P. thunbergii* forest (PPF), mixed forest (MPF + MLF), and pure *Liquidambar* forest (PLF) using environmental factors as explanatory variables during forest succession.

The three forest types shared 13.5% of the total OTUs (765) (Fig. 4). The number of unique OTUs in each forest increased during the succession process; the PPF site had the lowest number of unique OTUs (347, 6.1%), and the PLF site harbored the highest number of unique OTUs (700, 12.4%) (Fig. 4). Distance-based redundancy analysis (dbRDA) based on the OTU data showed that the three forest types formed distinct fungal community structures, and subsequent permutational multivariate analysis of variance (PERMANOVA) confirmed the difference among the communities (*P* < 0.05 in all pairs) (Fig. 5a). Interestingly, the fungal community structure in the mixed forest between MPF and MLF did not differ. The environmental factors accounted for 15.4% of the total variance and 39.2% of fitted for fungal communities on dbRDA1 as well as for 5.5% of total variation and 13.9% of fitted for fungal communities on dbRDA2. The distance-based linear model (DistLM) using the environmental factors as explanatory variables showed that the tested environmental factors were significantly correlated with fungal communities (*P* < 0.05 for each of the variables) (Fig. 5a). The soil pH and soil organic carbon (SOC), as well as the ratio of fungal and bacterial biomass (F:B) were positively correlated with the fungal community in the mixed sites (MLF and MPF). The fungal biomass (FB) and the activity of acid phosphatase (PHO) were significantly correlated with the fungal community in the PPF site. The activity of cellobiohydrolase (CEL) was significantly correlated with the fungal community in the PLF site.

### Fungal community structure of predicted function during forest succession.

**FIG 6:**
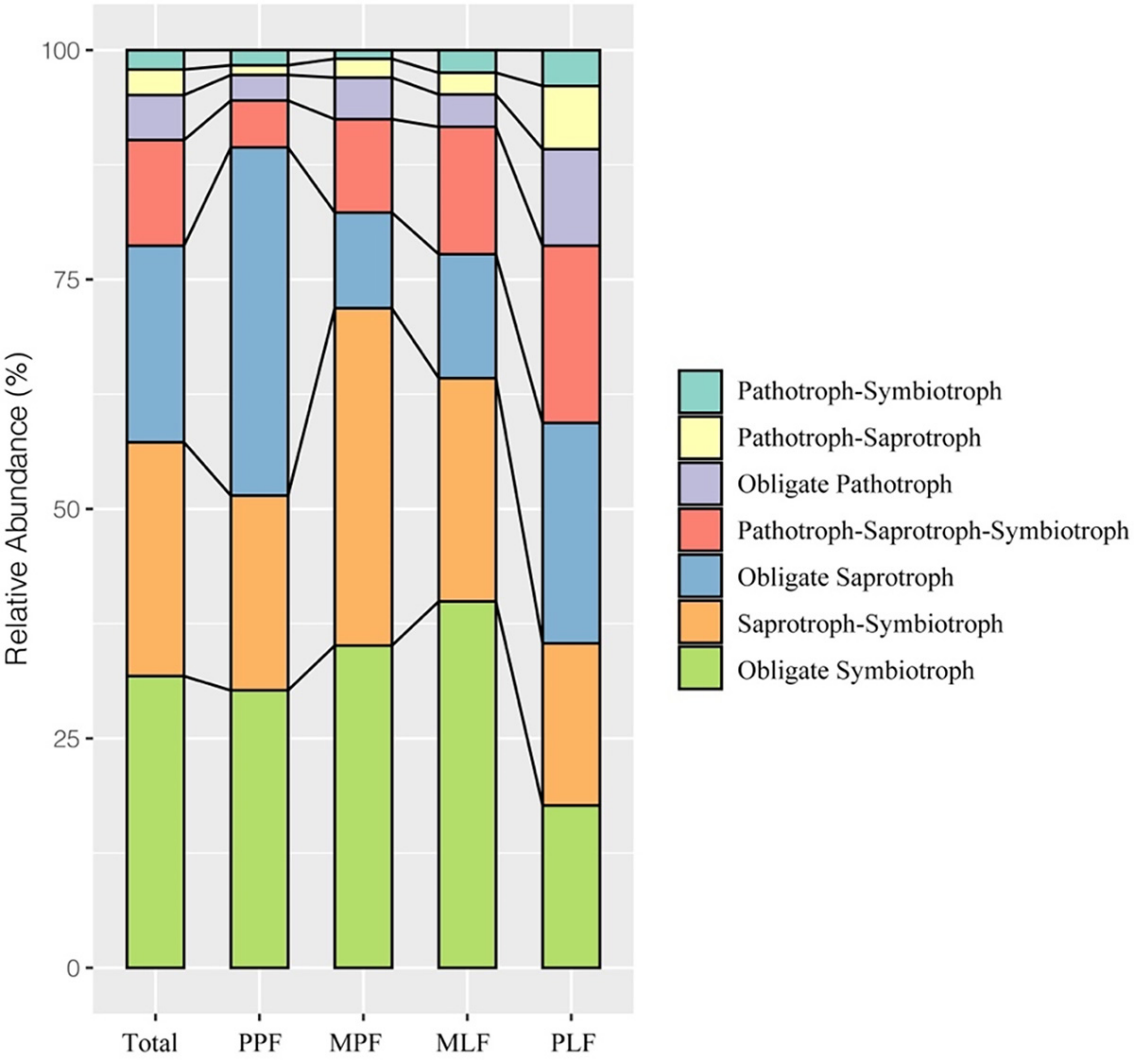
The fungal functional composition in pure *P. thunbergii* forest (PPF), mixed forest (MPF + MLF), and pure *Liquidambar* forest (PLF) during forest succession.

In total, 2,304 OTUs were included in the FUNguild analysis. The symbiotrophs comprised the most dominant trophic mode, covering 31.8% of assigned sequences, followed by the saprotrophs-symbiotrophs (25.5%), saprotrophs (21.4%), pathotrophs-saprotrophs-symbiotrophs (11.5%), pathotrophs (4.9%), pathotrophs-symbiotrophs (2.8%), and pathotrophs-saprotrophs (2.1%) (Fig. 6). During the succession, the abundance of symbiotrophs increased in the mixed forest (MPF and MLF) and decreased significantly in the PLF (*P* < 0.01). The abundance of saprotrophs-symbiotrophs increased in the MPF and then decreased sharply in the mixed and pure *Liquidambar* forest (MLF and PLF). The abundance of saprotrophs differed among the three forest types (*P* < 0.01), in which the PPF had the highest abundance and the mixed forests (MPF and MLF) had the lowest abundance. The pathotrophs had the lowest abundance in the PPF site and increased significantly after the succession (*R*^2^ = 0.661). Similarly, the abundances of pathotrophs-symbiotrophs, pathotrophs-saprotrophs, and pathotrophs-saprotrophs-symbiotrophs also increased during forest succession (*R*^2^ = 0.704, *R*^2^ = 0.785, *R*^2^ = 0.995, respectively). Some specific functional groups (guilds) significantly differed in abundance among forests during succession (see Fig. S3 and S4 in the supplemental material).

Distance-based redundancy analysis based on the functional groups showed that the three forest types formed separated functional structures (Fig. 5b). Subsequent PERMANOVA confirmed the differences in functional structures (*P* < 0.05 in all pairs). However, the functional structures did not differ in a mixed forest between MPF and MLF. The DistLM analysis showed that the soil pH, SOC, total nitrogen (TN), F:B, CEL, GLR, and SUL were significantly correlated with the functional structure in the mixed forest (*P < *0.05), whereas the enzyme activities of PHO and fungal biomass were significantly corrected with the PPF site (*P* < 0.05). The activities of CEL, GLS, and fungal biomass were significantly correlated with the PLF site (*P* < 0.05) (Fig. 5b).

## DISCUSSION

### Soil fungal biomass, enzyme activity, and fungal α-diversity during forest succession.

We observed significant differences in fungal biomass among the three forests. The fungal biomass is a determinant of enzyme activities in forest soil (32) and contributes to total microbial biomass (33). Increased fungal biomass could indicate faster litter composition in the soil (34), affecting fungal biomass (35). The fungal biomass in the pure *L. formosana* forest (PLF) was significantly higher than that in the other sites, which is in line with a previous study that suggested that the deciduous forest has a significantly higher soil fungal biomass than that of the conifer forest (15). The discrepancies in changes in aboveground plants could partly contribute to this observation. The litter input in pure *L. formosana* forest contains more easily available nutrients for fungi than the pure pine forest. The contents of fungal biomass increased during forest succession, which may indicate the faster decomposition rate due to the shift from conifer to broadleaved trees.

The shift in aboveground plant species caused by forest succession can affect the plant residue biochemistry and the amount and stability of the soil C pool via changing the aggregate allocation of carbon (36, 37). In this study, the enzyme activities in C, N, and S cycles increased during forest succession. The enzyme involved in the C cycle showed higher activities in the PLF forest than those in the other forests, positively correlated with soil fungal biomass. The PPF forest had a lower soil pH with a low soil organic carbon content than that of the other forests, which may explain the lower enzyme activity in the C cycle in the PPF forest. Different tree types can affect the ratio of soil fungi and bacteria by increasing the biomass of fungi over bacteria, which can alter soil enzyme activities (38). This might also explain the difference in enzyme activities among the three forests. In our study, the *N*-acetylglucosaminidase (NAG) and sulfatase activities were significantly higher in the PLF site. NAG is involved in the decomposition of chitin and fungal mycelium (39), and sulfatase is involved in the hydrolyzation of sulfate esters (40). Previous studies have indicated that the activities of both enzymes were correlated positively with soil microbial biomass (38, 41). The NAG and sulfatase activities being significantly higher in the PLF may result from the increased fungal biomass, which can also explain the significantly increased fungal α-diversity and richness in the PLF site. Moreover, microbes under low P availability might be forced to release phosphatase to meet the demand of P for further growth (42), which causes the higher PHO activity in the initial pure pine forest.

### Soil fungal community structure at OTU and taxonomic levels during forest succession.

The three forests during the succession formed distinct soil fungal communities in our study. The plant species can strongly affect the soil microbial community structure by allocating plant C and other nutrients (8, 9). The variation in tree species in the three forest types could be the key contributor to the observation. The physiochemical properties of soil (e.g., soil pH and nutrient availability) can be altered by tree species (41), which are important regulators affecting soil microbial communities (10, 11). Soil pH and soil C and N content also significantly differed among the three forests during the succession in our study (see Table S1 in the supplemental material). Interestingly, the fungal community structures in the mixed forest between MPF and MLF did not differ. A similar result was observed for the bacterial community in the same sites in our previous study (31). A previous study has also found that forest type strongly impacts microbial community structure at nutrient-poor sites but less at nutrient-rich sites (43). Therefore, further studies are necessary to elucidate the possible reasons.

Ascomycota, Basidiomycota, and Mortierellomycota were the dominant phyla during forest succession, which is consistent with the results of previous studies where they were the main soil fungal groups (44). During forest succession, the abundance of Ascomycota increased, whereas that of Basidiomycota decreased with the accumulation of soil nutrients. Following forest vegetation restorations, similar results were obtained due to different land uses on the Loess Plateau (45). *Mortierella* and *Penicillium* were the dominant genera during forest succession, reflecting the changes in the availability of nutrients and competition between species. *Penicillium* can degrade cellulose in the early stages of decomposition (46). *Mortierella* spp. can transform phosphorus from an insoluble to a soluble form for plant uptake (47).

The soil properties as explanatory variables were all correlated with the fungal community structures in the study. Previous studies have shown that SOC, soil pH, and TN are factors influencing the fungal community structure (48, 49). Among these, soil pH was one of the essential variables, which showed a positive correlation with Ascomycota fungi (50, 51). However, we did not detect a correlation between the abundance of Ascomycota and soil pH. The range of soil pH in our study was small, which may be attributed to fungi being less sensitive to pH changes than bacteria (52). This could be the reason why it was difficult to ascertain such a correlation.

### Fungal community structure of predicted function during forest succession.

Functionally, the fungal trophic modes (saprotroph, symbiotroph, and pathogen) showed different trends during the succession. The abundance of saprotrophs in the mixed forest (MPF and MLF) was significantly lower than that in the pure forests (PPF and PLF). The abundance of saprophytic fungi can be linked to the plant tree species, most likely due to the difference in the biochemistry of litter input, and the mixed litter may inhibit the degradation process of saprophytic fungi (53). The abundance of symbiotrophs was higher in the mixed forests than that in the pure forests. The mixed forests in our study had higher abundance of ectomycorrhizal (ECM) fungi and SOC content than other forests, which is in line with a previous finding that soil organic matter has a positive correlation with ECM fungi (54, 55). Pathotrophic fungi are suspected of causing diseases or negatively affecting plant performance because they derive nutrient substances by attacking host cells (56). Despite being less abundant during forest succession, the relative abundance of pathotrophs increased during succession. The weakened tree caused by pine wilt disease could increase the occurrence of needle disease and pathogens (57). A previous study indicated that the changes in pathogens could indicate a complex interaction between soil and plants, and increased vegetation, for example, has increased the heterogeneity of the litter, which can provide diverse ecological niches for pathogens (58).

### Conclusion.

The forest succession induced by pine wood nematode disease significantly increased the fungal biomass, soil enzyme activity, and fungal diversity. The forests in different stages of succession formed a distinct fungal community and functional structures in which the fungal community shifted from Basidiomycota dominance in the initial conifer forest to a higher abundance of Ascomycota in the eventual broadleaved forest. Moreover, the symbiotrophs were the most dominant group and the abundance of pathotrophs increased during the succession. Functionally, saprotrophs, symbiotrophs, and pathotrophs dominated the fungal community in the initial forest, mixed forest, and eventual forest, respectively. The soil pH and SOC were the most important factors affecting the fungal community and functional structure, indicating the importance of environmental factors in shaping microbial community. These findings provide new insight into the responses of microbes to disease-induced forest succession and also highlight the importance of the linkage between plants and microbes in the forest ecosystem.

## MATERIALS AND METHODS

### Study sites and soil sampling.

The study sites are in Nanjing Zijin Mountain Park, Nanjing, Jiangsu Province, China. The mountain area covers approximately 4,500 ha with a latitude of 32°03′22.73′N and longitude of 118°51′26.07′E. The altitudes above sea level at the top and foot of the mountain are approximately 449 and 20 m, respectively. This region belongs to the transitional zone of the subtropical monsoon climate. The annual average precipitation is 1,000 mm, and the average sunshine hours are approximately 2,213 h per year. The annual mean temperature is 15.4°C, ranging from 40.7°C in August to −14.0°C in January (59).

The soil samples were collected in November 2017, and more details were described previously (31). Briefly, three forest types were chosen, including one pure *P*. *thunbergii* forest (PPF), one pure *L*. *formosana* forest (PLF), and one mixed forest of *P*. *thunbergii* and *L*. *formosana* (MPF and MLF). The ratio of *P*. *thunbergii* and *L*. *formosana* trees in mixed forest is about 1:1. The ground vegetations are mainly shrubs in each forest, e.g., Serissa japonica (Thunb.) in in the PPF site, Symplocos paniculata and Lindera glauca in mixed forest, and Smilax china and Smilax glaucochina in PLF forest. Each forest type had three plots with dimensions of 50 m by 50 m each and were situated 500 m apart from each other. Five trees of *P*. *thunbergii* or *L*. *formosana* were randomly chosen in each plot. The soil was collected at a depth of 0 to 10 cm after litter removal in three directions with a 120° angle around each tree and mixed as one sample, resulting in 60 samples in total (15 samples per pure forest + 30 samples in mix forest). The soil samples were placed on ice and delivered to the laboratory for further analysis.

### Soil enzyme activity analysis.

Seven commonly reported soil enzyme activities related to C, N, S, and P cycles were determined. The enzymes involved in the C cycle included β-xylosidase (XYL), β-d-glucuronidase (GLR), β-cellobiosidase (CEL), and β-glucosidase (GLS), and those involved in N, P, and S cycles included *N*-acetylglucosaminidase (NAG), phosphatase (PHO), and sulfatase (SUL), respectively. The soil enzyme activity was measured fluorometrically with a 96-well plate using 4-methylumbelliferone-linked (4-MUB) enzyme substrates following a previously described method (60). Briefly, 2 g of soil sample (fresh weight) was added into a 30-mL acetate buffer solution (50 mM with pH 5) in a 50-mL centrifuge tube. The soil suspensions were shaken at 180 rpm for 40 min at 25°C to break large soil particles followed by the addition of 170 mL sodium acetate solution to 200 mL. The 200 μL suspension was added into each well of the 96-well plate followed by the addition of 50 μL of 4-MUB-linked substrate (200 mM) to start the reaction. Each soil sample had four replicates. For each replicate, four reactions were also included as follows: blanks (200 μL substrate with 50 μL double-distilled water), quench standards (200 μL substrate with 50 μL 4-MUB), negative controls (200 μL buffer solution with 50 μL 4-MUB-linked substrate), and reference standards (200 μL buffer solution with 50 μL 4-MUB). Then, 10 μL NaOH (1 M) was added to each well to cease the reaction after incubation for 4 h at 25°C in the dark. The fluorescence was measured using a microplate fluorometer with 365-nm excitation and 450-nm emission filters. The enzyme activities were expressed as MUB released in nmol per gram dry soil per hour (nmol · g^−1^ · h^−1^).

### DNA extraction, amplification of ITS rRNA region, Illumina Miseq. Sequencing, and qPCR for fungal biomass.

The soil genomic DNA was extracted from 0.3 g fresh soil using the Power Soil DNA isolation kit (OMEGA BIO TEK, Norcross, GA, USA) following the manufacturer's instructions. The extracted DNA was measured using a NanoDrop 1000 spectrometer (NanoDrop Technologies, Wilmington, DE, USA) and checked by gel electrophoresis. The DNA product was subjected to the PCR amplification of the internal transcribed spacer 1 (ITS1) region using the primer set ITS1F (5′-CTTGGTCATTTAGAGGAAGTAA-3′) and ITS2R (5′-GCTGCGTTCTTCATCGATGC-3′) with a thermocycler PCR system (GeneAmp 9700, ABI, USA). The PCRs were performed in triplicate in a 20-μL mixture containing 4 μL of 5× FastPfu buffer, 2 μL of 2.5 mM deoxynucleoside triphosphates (dNTPs), 0.8 μL of each primer (5 μM), 0.4 μL of FastPfu polymerase, 0.2 μL of bovine serum albumin (BSA), and 10 ng of template DNA, and the remaining volume was replenished with double-distilled water. The PCRs were conducted using the following program: 3 min of denaturation at 95°C, 36 cycles of 30 s at 95°C, 30 s for annealing at 55°C, 45 s for elongation at 72°C, and a final extension at 72°C for 10 min. The PCR products were checked and extracted from a 2% agarose gel and further purified using the AxyPrep DNA gel extraction kit (Axygen Biosciences, Union City, CA, USA) and quantified using QuantiFluor-ST (Promega, USA) according to the manufacturer’s protocol. The purified amplicons were subjected to sequencing with a paired end (PE = 300) on the Illumina MiSeq platform (Illumina, San Diego, USA) according to the standard protocols at Majorbio Bio-Pharm Technology Co. Ltd. (Shanghai, China).

The fungal biomass (FB) in the soil was detected by quantitative PCR (qPCR). The fungal 18S rRNA genes were amplified using the specific-primer pair FF390 (5′-ATTACCGCGGCTGCTGG-3′) and FR1(5′-AIC-CATTCAATCGGTAIT-3′) (61). The qPCR was executed using a Bio-Rad CFX96 iCycler on 96-well white-welled polypropylene plates (62). The 20 μL reaction mixture contained a 1× SsoAdvanced universal SYBR green supermix (Bio-Rad, USA), 3 ng of template DNA, 250 nM forward primer, and 250 nM reverse primer. The reaction condition was as follows: initial denaturation at 95°C for 3 min, denaturation at 95°C for 15 s, and combined annealing and extension at 60°C for 1 min over 40 cycles. The melting curve analysis was conducted after PCR was run by raising the temperature from 65 to 95°C (0.5°C per 5 s).

The stand curves were conducted using DNA extracted from Phlebia radiata FBCC43 (genome size 40.92 Mb; FBCC culture collection, University of Helsinki, Finland). The copy number of the 18S rRNA gene was known (63). The fungal biomass in each sample was calculated based on the standard curve described previously (63).

### Sequence data processing and statistical analysis.

The paired-end sequences were processed using Mothur software (version 1.44.3) following the standard operating procedure (SOP) with some modifications (64, 65) The sequences were screened out if they contained ambiguous (N) bases, homopolymers longer than 8 bp, average quality score lower than 25, putative chimeras (using UCHIME in Mothur), and fewer than 250 bp. The amplification errors were checked using the PCR.seq command. The sequences were pairwise aligned using the align.seq command and preclustered with two base pair differences to remove sequences that were likely due to sequencing errors (66). The high-quality and unique sequences were classified against the UNITE database (version 8) with the bootstrap cutoff as 80 (67). The remove lineage command removed nonfungal sequences. The fungal sequences were then clustered into operational taxonomic units (OTUs) with 97% similarity using the average neighbor-joining algorithm. All global singletons (OTUs containing only one sequence across all samples) were omitted as being of uncertain origin (68). The fungal community species richness (Sobs), α-diversity (Shannon), and species evenness (Shannoneven) were calculated by subsampling the sequences with the minimum size of the sample among all samples.

The OTUs were assigned into functional groups using the FUNGuild database, in which the fungi were divided into three trophic modes, namely, pathotrophs, symbiotrophs, and saprotrophs (69). Within these trophic modes, fungi were further divided into a total of 21 categories, broadly referred to as guilds. For example, the pathotrophs include plant pathogens, animal pathogens, and fungal parasites. The symbiotrophs include arbuscular mycorrhizal fungi, ectomycorrhizal fungi (ECM), ericoid mycorrhizal fungi (ErM), and endophytes. The saprotrophs include plant saprotrophs, dung saprotrophs, litter saprotrophs, undefined saprotrophs, and wood saprotrophs.

One-way analysis of variance by Duncan’s tests was used to determine the significant differences (*P *< 0.05) in fungal α-diversity, fungal biomass, and enzyme activities among samples in SPSS 21.0 for Windows (SPSS Inc., Chicago, USA). Distance-based redundancy analysis (dbRDA) and permutational multivariate analysis of variance (PERMANOVA) were used to visualize and detect the difference in fungal community and functional structure based on the relative abundance of OTUs or guilds (70). A distance-based linear model (DistLM) was used to test the correlation between the fungal community or functional structure and the environmental variables. The soil properties in the same site from the same project were examined (see Table S1 in the supplemental material). The DistLM analyses were performed in PRIMER 7+ (71). The linear discriminant analysis (LDA) coupled with effect size (LEfSe) (http://huttenhower.sph.harvard.edu/galaxy) was used to identify the fungal taxa or FUNGuild differentially represented between samples (72). The LEfSe was performed with an average relative abundance of taxa of >0.001 to hold as many taxa as possible for meaningful comparisons and remove all rare taxa in the analysis. The criteria for the significant difference of fungal taxa and function guilds were set up with LDA > 3 and *P *< 0.05. The default value (LDA = 2.0) is widely accepted in LEfSe analysis. However, the higher value of LDA can reduce the number of differences in taxa or functional groups and increase the analysis accuracy (73).

### Data availability.

The data sets generated during and/or analyzed during the current study are available in the NCBI Sequence Read Archive (SRA) database (https://www.ncbi.nlm.nih.gov/sra) under accession number PRJNA580298.
