# Supplementary material for: Soil Fungal Community Structure and Function Shift during a Disease-Driven Forest Succession
Source: Microbiol Spectr. 2022 Sep 8;10(5):e00795-22. doi: 10.1128/spectrum.00795-22 (PMC9602832; doi:10.1128/spectrum.00795-22)
Supplement: Supplemental file 1 — Supplemental material. Download spectrum.00795-22-s0001.pdf, PDF file, 0.5 MB [file spectrum.00795-22-s0001.pdf]

Supplementary files:

Table S1*. The soil physicochemical properties in the three forests during forest succession.

Succession process	Forest type	Soil organic carbon (%)	Soil pH	Total nitrogen (%)	Soil moisture (%)	Microbial biomass carbon (mg/kg)	Bacterial biomass(copy/kg)
**Initial coniferous forest**	**Pure pine**	4.77±1.20 [a]	4.91±0.32 [a]	0.08±0.01 [a]	14.96±8.46 [a]	66.91±29.67 [a]	6056.22± 1497.70 [ab]
**Gradual mixed forest**	**Mixed pine**	6.88±0.96 [b]	5.41±0.25 [b]	0.11±0.03 [a]	16.99±3.14 [a]	60.44±32.30 [a]	7356.75± 2418.55 [a]
	**Mixed Liquidambar**	6.89±1.73 [b]	5.41±0.32 [b]	0.13±0.04 [b]	19.59±5.41 [a]	72.54±23.00 [a]	5375.72± 1887.20 [b]
**Eventual broadleaved forest**	**Pure Liquidambar**	6.04±1.26 [a]	5.58±0.11 [b]	0.15±0.01 [b]	18.36±11.93 [a]	79.28±30.70 [a]	5273.05± 1140.67 [b]

Letters in the table show the significant difference ($P < 0.05$) in each of the properties (columns) among the three forests.

*Table from Qu et al. (2020) in the same project.

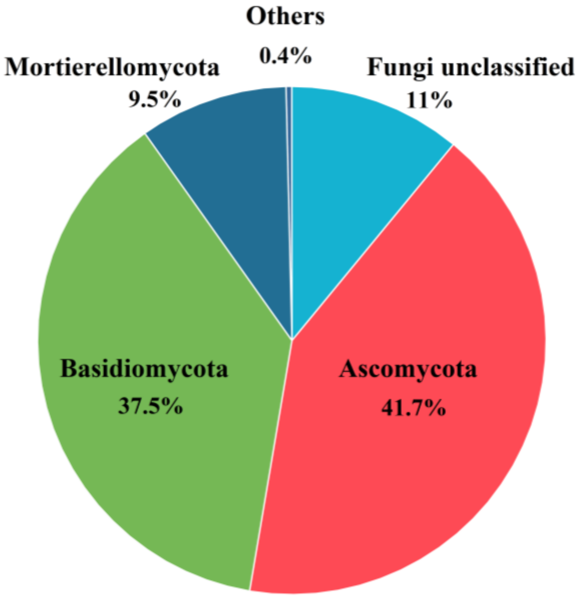

Figure S1. Pie chart showing the overall distribution of fungal sequences (n = 2,863,208) at phylum levels.

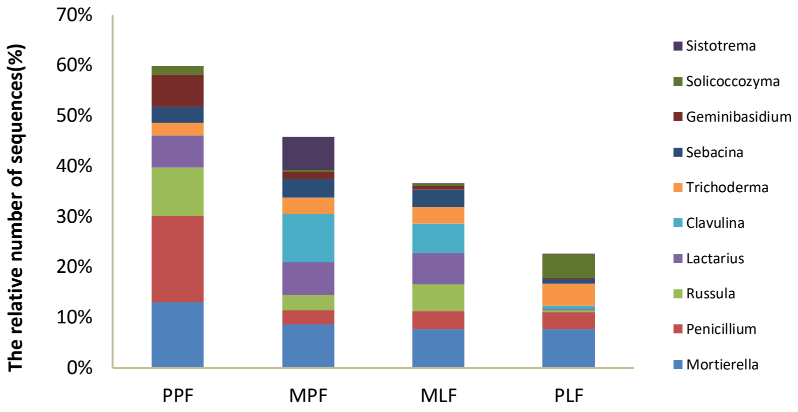

Figure S2. The most abundant fungal groups at the genera level (top 10) in pure *P. thunbergii* forest (PPF), mixed forest (MPF + MLF), and pure *Liquidambar* forest (PLF) along forest succession.

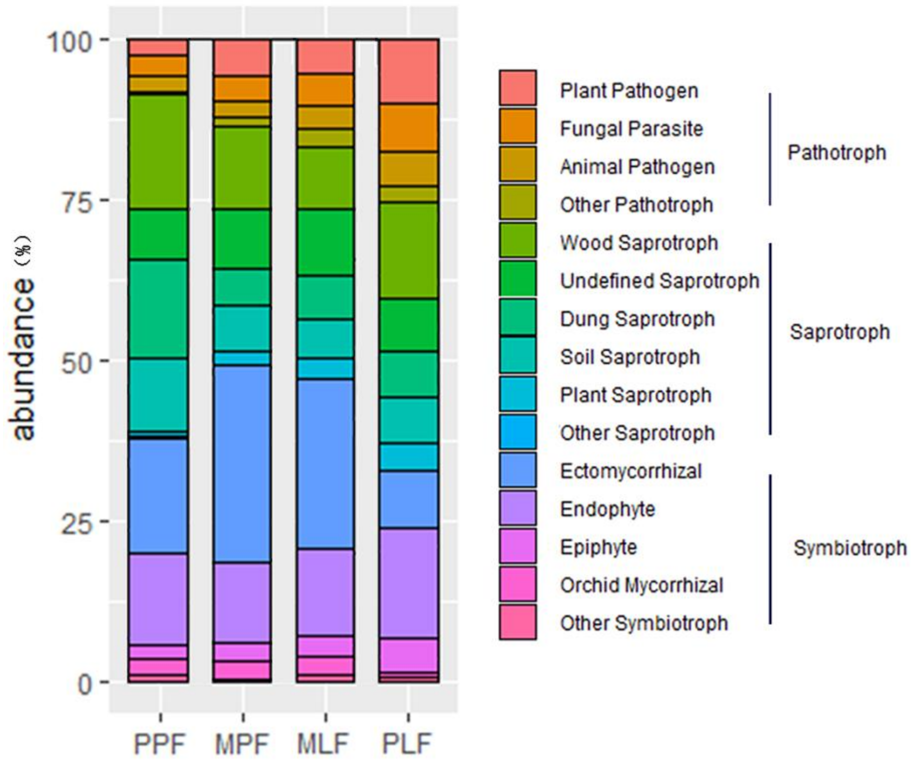

Figure S3. The fungal functional composition at the guild level in pure *P. thunbergii* forest (PPF), mixed forest (MPF + MLF), and pure *Liquidambar* forest (PLF) during forest succession.

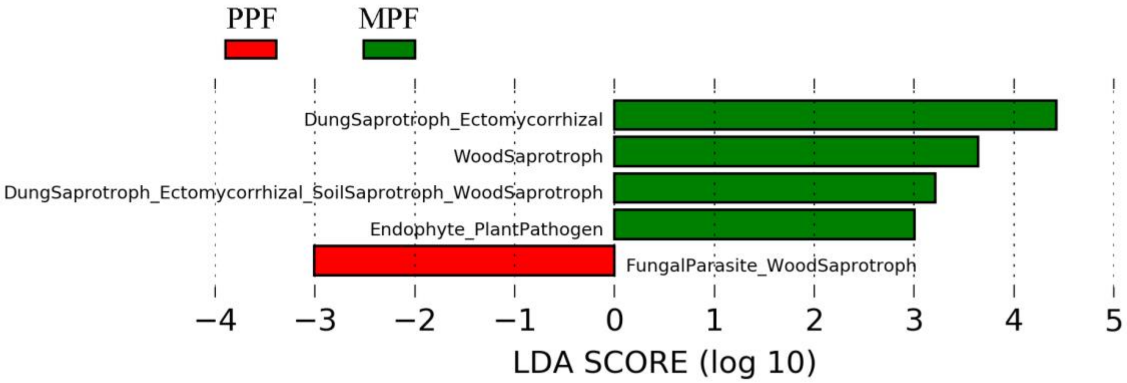
PPF
MPF
DungSaprotroph_Ectomycorrhizal
WoodSaprotroph
DungSaprotroph_Ectomycorrhizal_SoilSaprotroph_WoodSaprotroph
Endophyte_PlantPathogen
FungalParasite_WoodSaprotroph
−4 −3 −2 −1 0 1 2 3 4 5
LDA SCORE (log 10)

(a)

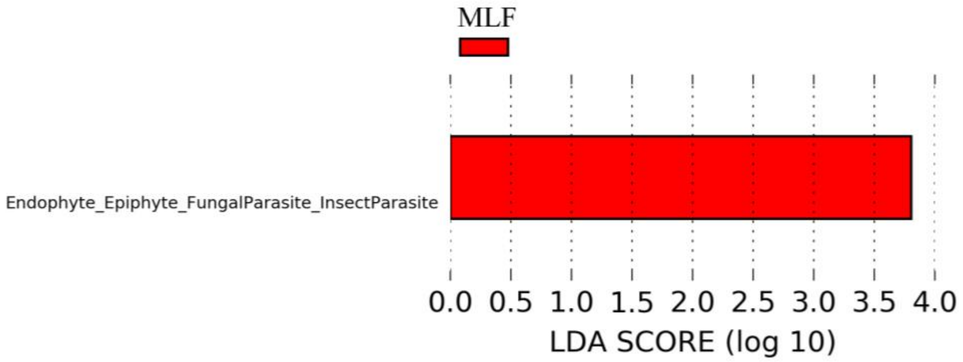
MLF
Endophyte_Epiphyte_FungalParasite_InsectParasite
0.0 0.5 1.0 1.5 2.0 2.5 3.0 3.5 4.0
LDA SCORE (log 10)

(b)

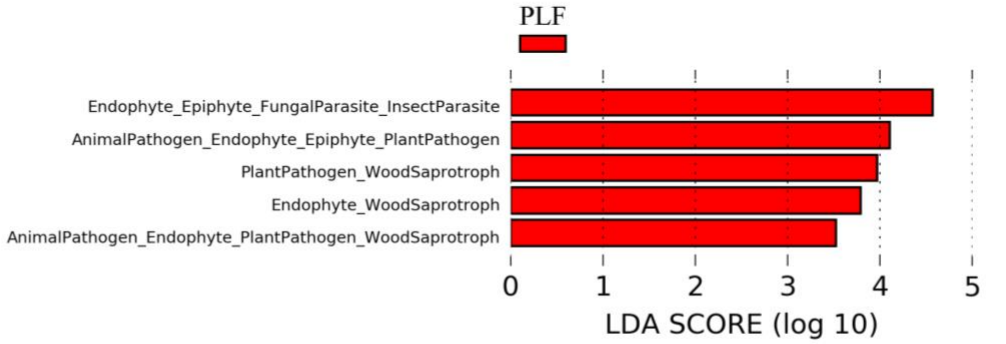

(c)

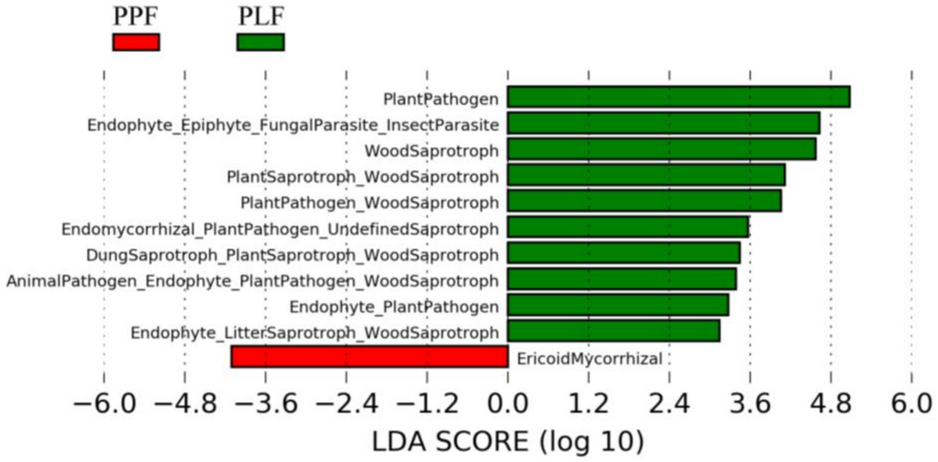

(d)

Figure S4. LEfSe analysis showing that the fungal functional at the guild level significantly differed between the mixed and pure *P. thunbergii* forest (MPF + PPF) (a); the mixed forest (MPF + MLF) (b); the mixed and pure *Liquidambar* forest (MLF + PLF) (c) during the succession; and the pure *P. thunbergii* forest and pure *Liquidambar* forest (PPF + PLF).
